# The occurrence and molecular detection of *mcr-1* and *mcr-5* genes in *Enterobacteriaceae* isolated from poultry and poultry meats in Malaysia

**DOI:** 10.3389/fmicb.2023.1208314

**Published:** 2023-08-03

**Authors:** Md Rezaul Karim, Zunita Zakaria, Latiffah Hassan, Nik Mohd Faiz, Nur Indah Ahmad

**Affiliations:** ^1^Department of Veterinary Pathology & Microbiology, Faculty of Veterinary Medicine, Universiti Putra Malaysia, UPM, Serdang, Selangor, Malaysia; ^2^Bangladesh Livestock Research Institute, Dhaka, Bangladesh; ^3^Institute of Bioscience, Universiti Putra Malaysia, UPM, Serdang, Selangor, Malaysia; ^4^Department of Veterinary Laboratory Diagnostics, Faculty of Veterinary Medicine, Universiti Putra Malaysia, UPM, Serdang, Selangor, Malaysia; ^5^Department of Veterinary Clinical Studies, Faculty of Veterinary Medicine, Universiti Putra Malaysia, UPM, Serdang, Selangor, Malaysia

**Keywords:** *Enterobacteriaceae*, MCR, colistin resistance, poultry, Malaysia

## Abstract

The advent of antimicrobials-resistant (AMR), including colistin-resistant bacteria, poses a significant challenge to animal and human health, food safety, socio-economic growth, and the global environment. This study aimed to ascertain the colistin resistance prevalence and molecular mechanisms of colistin resistance in *Enterobacteriaceae*. The colistin resistance was determined using broth microdilution assay, PCR; and Sanger sequencing of *mcr* genes responsible for colistin resistance in *Enterobacteriaceae* (*n* = 627), including *Escherichia coli* (436)*, Salmonella* spp. (*n* = 140), and *Klebsiella pneumoniae* (*n* = 51), obtained from chicken and chicken meats. Out of 627 *Enterobacteriaceae*, 8.6% of isolates exhibited colistin resistance phenotypically. Among these colistin resistant isolates, 9.3% (*n* = 37) were isolated from chicken meat, 7.2% (*n* = 11) from the cloacal swab of chicken and 7.9% (*n* = 6) from the litter samples. Overall, 12.96% of colistin-resistant isolates were positive with *mcr* genes, in which *mcr-1* and *mcr-5* genes were determined in 11.11% and 1.85% of colistin-resistant isolates, respectively. The *E. coli* isolates obtained from chicken meats, cloacal swabs and litter samples were found positive for *mcr-1*, and *Salmonella* spp. originated from the chicken meat sample was observed with *mcr-5*, whereas no *mcr* genes were observed in *K. pneumoniae* strains isolated from any of the collected samples. The other colistin resistance genes, including *mcr-2, mcr-3, mcr-4, mcr-6, mcr-7, mcr-8, mcr-9,* and *mcr-10* were not detected in the studied samples. The *mcr-1* and *mcr-5* genes were sequenced and found to be 100% identical to the *mcr-1* and *mcr-5* gene sequences available in the NCBI database. This is the first report of colistin resistance *mcr-5* gene in Malaysia which could portend the emergence of *mcr-5* harboring bacterial strains for infection. Further studies are needed to characterize the *mr-5* harbouring bacteria for the determination of plasmid associated with *mcr-5* gene.

## Introduction

Antimicrobial agents are essential medicines in animals and humans to curb infections. Owing to the overuse and abuse of antimicrobial agents, the globe is being faced with the rapid proliferation of resistant microbes. Currently, the advent of antimicrobial-resistant (AMR) bacteria poses a significant challenge to animal and human health, food safety, socio-economic growth, and the global environment (Theuretzbacher, 2017). The proliferation of Gram-negative bacterial strains which are resistant to multiple drugs, and the absence of new drugs to combat such microbes, reintroduced colistin as a last-line therapy (Ezadi et al., 2019).

The resistance to colistin is a crucial problem to be tackled today. Numerous studies have demonstrated that this colistin resistance was present in various bacterial strains around the world. The plasmid-mediated *mcr* genes are accountable for exceptional colistin resistance, as it is a conduit that spreads via horizontal transmission from one bacterial strain to another and through food chain or direct contact to humans, animals, and the environments (Gharaibeh and Shatnawi, 2019). Before 2015, all documented colistin-resistance was chromosomally regulated, involving modification of a two-component regulatory structure, phoPQ and pmrAB with the negative regulator, *mgrB* gene alteration (Cannatelli et al., 2013; Liu et al., 2016). In 2016, the *Escherichia coli* strains isolated from humans, retail chicken meat and pork, and *Klebsiella pneumoniae* strains isolated from humans were reported with the plasmid-encoded *mcr-1* gene in China (Liu et al., 2016). The plasmid-mediated new *mcr* genes have rapidly emerged. The therapeutic effectiveness of colistin has been compromised by the advent of the plasmid-encoded *mcr* genes, including *mcr-1* to *mcr-10,* which were reported during the last four years (Ling et al., 2020). The *E. coli* isolates recovered from chicken liver, and chicken feed in the trough (Yu et al., 2016), and poultry meat in Malaysia were found positive for *mcr-1* gene (Aklilu and Raman, 2020).

Poultry meat is an important source of protein for humans, it could also be a significant conduit for spreading multidrug-resistant bacterial species from food-producing animals to humans. Previous study has shown that retail chicken meat plays a role in disseminating multiple antibiotic-resistant strains among humans and their environment, posing a severe threat to environmental health and food safety (Aidara-Kane et al., 2013). The colistin resistance-producing gene, *mcr-1*, was present in 52.1 percent of the *E. coli* isolates from raw chicken meat (Aklilu and Raman, 2020). Colistin resistance was found in more in 36.4% of bacteria from poultry chicken and 20% of strains isolated from native chicken in Bangladesh (Islam et al., 2020). In Nepal, it was reported that 27 (22.8%) of colistin-resistant *E. coli* in broiler farms carried the *mcr-1* gene (Joshi et al., 2019). Aside from that, the horizontal transmission is thought to be the main mechanism for the spread of colistin resistance *mcr* genes in *Enterobacteriaceae* worldwide (Gharaibeh and Shatnawi, 2019).

With potent *in vitro* transfer rates and frequently harboured alongside other resistance determinants like β -lactamases, *mcr* genes have been identified on a variety of conjugative plasmids (IncI2, IncHI2, IncX4, and pHNSHP45) (EMA (European Medicine Agencey), 2016). As the mobilized colistin resistance (*mcr*) genes driven by plasmids are quickly emerging, appropriate information on colistin resistance, including the incidence and epidemiological studies of *mcr*-positive cases, is required to apply steps to prevent and manage its dissemination. Therefore, the aim of the study was to determine the prevalence and molecular determinants underlying colistin resistance in *Enterobacteriaceae* (*E. coli, Salmonella* spp., and *Klebsiella pneumoniae*) isolates recovered from poultry and poultry meats in Malaysia.

## Materials and methods

### Ethics approval

The ethical board of Universiti Putra Malaysia (UPM), Institutional Animal Care and Use Committee (IACUC) approved the research study protocol for collecting cloacal swabs from live poultry (UPM/IACUC/AUP-R091/2019).

### Study design and samples

**Figure 1:**
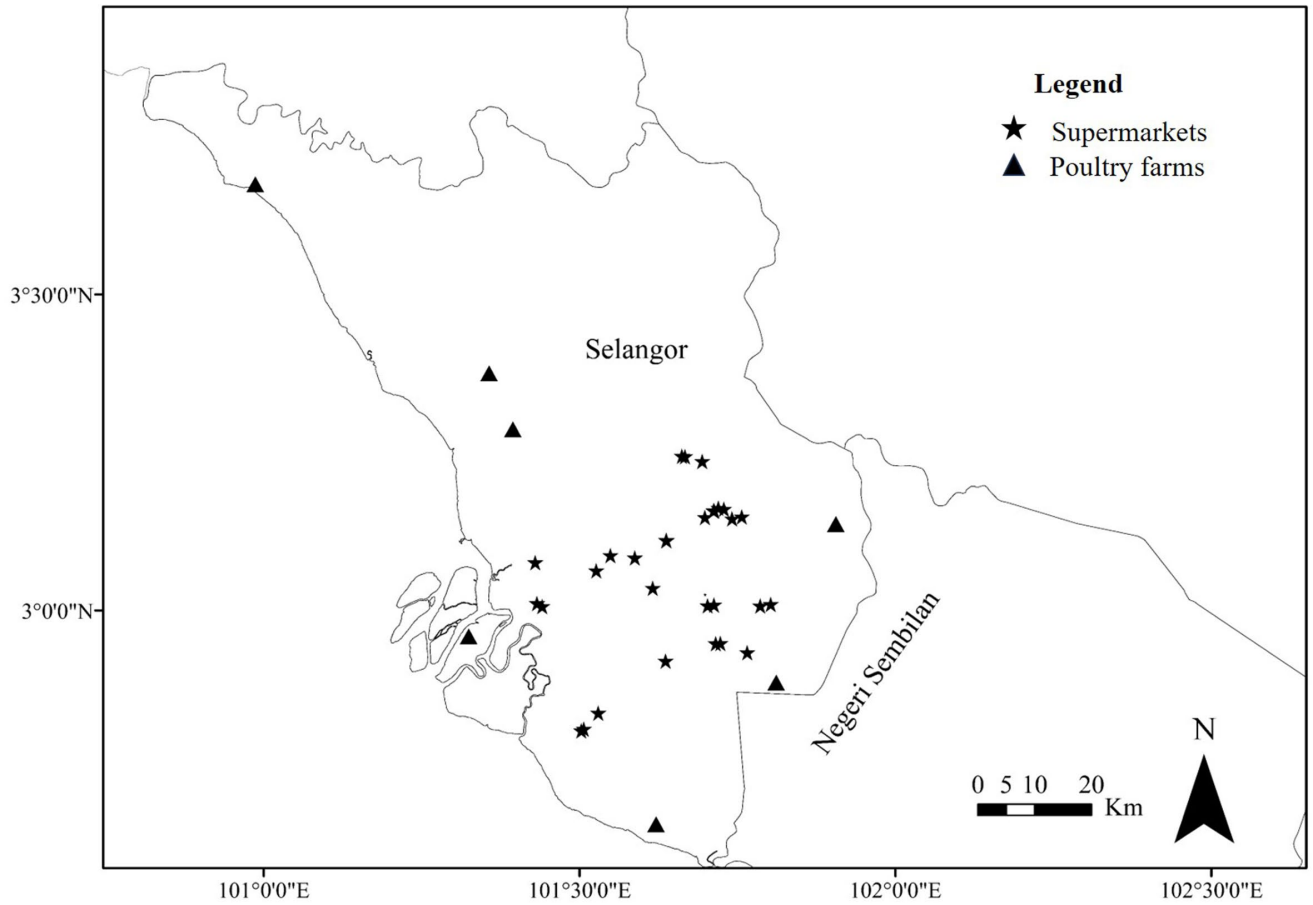
Map showing the sampling areas of Selangor, Malaysia. Star (*) and triangle (▲) marks indicating supermarkets and poultry farms, respectively. The geographic information software, ArcGIS v.10.4.1 for Windows was used to generate the sampling spot on the map.

The research study was performed in which chicken meat samples from supermarkets and cloacal swabs, and litter samples from chicken farms within Selangor, Malaysia (Figure 1) were collected from July 2019 to February 2021. Selangor is the densely populated area in Malaysia and most of the poultry farms are located in this state. Sterile plastic bags were used to collect the meat samples. The cloacal swab samples were collected aseptically from the healthy chicken and kept in sterile transport media, Stuart media. Litter samples were collected from the farms’ floors using a sterile spoon, and placed in a sterile plastic bag. In total, 543 samples, including 350 chicken meats, 144 cloacal swabs, and 49 litter samples (Table 1), were collected from supermarkets and poultry farms in different areas of Selangor in Malaysia (Figure 1). All collected samples were immediately transported in a sealed icebox to the Bacteriology Laboratory, Faculty of Veterinary Medicine at Universiti Putra Malaysia (UPM), Serdang, Selangor, Malaysia.

**Table 1:** List of the primers and sequences for the confirmation of *E. coli, Salmonella* spp., and *Klebsiella pneumoniae.*

Organisms	Gene	Primers	Primer sequence	Size (bp)	References
*E. coli*	16 s rRNA	ECO-1	GACCTCGGTTTAGTTCACAGA	585	Moawad et al. (2018)
		ECO-2	CACACGCTGACGCTGACCA		
*Salmonella* spp.	*invA*	139-F	GTGAAATTATCGCCACGTTCGGGCAA	284	Rahn et al. (1992)
		141-R	TCATCGCACCGTCAAAGGAACC		
*Klebsiella pneumoniae*	*mdh*	Pf	ATTTGAAGAGGTTGCAAACGAT	130	Ranjbar et al. (2016)
		Pr1	TTCACTCTGAAGTTTTCTTGTGTTC		

### Isolation and identification of bacterial strains

In the Enterobacteriaceae family, *Salmonella* spp., *E. coli,* and *K. pneumoniae* have public health importance and are more prevalent with colistin resistance *mcr* genes (Elbediwi et al., 2019). For this reason, *Salmonella* spp., *E. coli,* and *K. pneumoniae,* were isolated and identified phenotypically using the standard protocol of traditional cultural and biochemical tests (Ghafur et al., 2019; Sharma et al., 2019). In the pre-enrichment step, the samples were cultured in buffered peptone water (BPW, Oxoid, United Kingdom), including 25 g of meat in 225 mL, 10 g litter in 90 mL, and cloacal swabs in 10 mL BPW medium, samples were incubated at 37°C for 24 h. The meat samples with BPW were homogenized for 2 min in a stomacher. For *Salmonella* spp. isolation, 100 μL of homogenized BPW was mixed with 10 mL of Rappaport-Vassiliadis (RVS; Oxoid, United Kingdom). Then the RVS mixtures were incubated at 42°C for 24 h. One loop-full RVS of each sample was sub-cultured onto Xylose Lysine Deoxycholate agar (XLD; Oxoid, United Kingdom) and kept at 37°C for 24 h for incubation. On XLD, typically, Salmonella colonies were red with a black centre. One pure colony from XLD was cultured onto nutrient agar (NA, Oxoid, United Kingdom). For presumptive identification, biochemical tests (TSI, Urease, Citrate, and SIM) were performed with pure cultures grown onto NA. Serological confirmation of *Salmonella* spp. was performed using a slide agglutination test using Poly ‘O’ and ‘H’ antisera (Remel, United Kingdom). For *E. coli* and *K. pneumoniae* isolation, a loopful of suspension of BPW was inoculated onto MacConkey (Oxoid, United Kingdom). One presumptive colony of *E. coli* and *K. pneumoniae* were then sub-cultured onto NA and subjected to a standard biochemical test for their presumptive confirmation.

### Molecular confirmation of *Escherichia coli*, *Salmonella* spp., and *Klebsiella pneumoniae*

The genomic DNA was extracted from the pure culture of the isolates cultured on nutrient agar using the boiling and snap chill method (Pui et al., 2011). The phenotypically positive *E. coli*, *Salmonella* spp., and *Klebsiella pneumoniae* species were then confirmed with conventional PCR with species-specific gene primers (Table 1), in which positive controls included *E. coli* ATCC 25922, *Salmonella* ATCC 14028 and *Klebsiella pneumoniae* ATCC 700603 (Rahn et al., 1992; Ranjbar et al., 2016; Moawad et al., 2018).

### Colistin-susceptibility and minimum inhibitory concentration (MIC) assessment

Colistin-susceptibility and MIC in isolates were assessed by the ISO-20776 standard broth microdilution technique (BMD) jointly recommended by the EUCAST (2016a) with few modifications. In brief, a two-fold dilution (0.125–128 μg/mL) of the colistin sulfate salt (Sigma-Aldrich) was prepared on a 96 well microtitre plate. The bacterial inoculum was inoculated in each well with a final concentration of 5 × 10^5^ CFU/mL. Then the microtitre plate was incubated at 37°C for 16–20 h. After the incubation period, 30 μL of 0.015% resazurin solution was added to each well of the microtitre plate and incubated again at 37C for 1 h, during which the plate was routinely checked every 15 min. The colistin MICs were interpreted with the naked eye and recorded by observing the color change of resazurin (discoloration from blue to pink or purple indicates colistin resistance, while susceptibility is deduced when no color change- blue color). The test was performed in triplicates. During colistin-susceptibility testing, colistin-susceptible *E. coli* ATCC25922 and colistin-resistant (ColR) *E. coli* NCTC 13846 were utilized as negative and positive controls, respectively.

### Detection of the colistin resistance determinants, *mcr* genes

Colistin-resistant *Enterobacteriaceae* isolates were identified with MIC values greater than 2 μg/mL colistin (EUCAST, 2016b). The genomic DNA of the colistin-resistant (col-R) isolates was assessed with conventional PCR to detect colistin resistance (*mcr*) gene variants (*mcr-1* to *mcr-10*). According to previous studies, the detection of *mcr-1* to *mcr-5* (Rebelo et al., 2018) and *mcr-6* to *mcr-9* (Borowiak et al., 2020) was performed with multiplex PCR. The uniplex PCR was performed for *mcr-10* (Xu et al., 2021) by the previously designed oligonucleotide primers and the protocol (Table 2).

**Table 2:** List of the primers and sequences for the confirmation of *mcr* genes.

Set	Primers	Primer sequences	Size (bp)	References
Set-1	mcr-1F	AGTCCGTTTGTTCTTGTGGC	320	Rebelo et al. (2018)
	mcr-1R	AGATCCTTGGTCTCGGCTTG		
	mcr-2F	CAAGTGTGTTGGTCGCAGTT	715	
	mcr-2R	TCTAGCCCGACAAGCATACC		
	mcr-3F	AAATAAAAATTGTTCCGCTTATG	929	
	mcr-3R	AATGGAGATCCCCGTTTTT		
	mcr-4F	TCACTTTCATCACTGCGTTG	1,116	
	mcr-4R	TTGGTCCATGACTACCAATG		
	mcr-5F	ATGCGGTTGTCTGCATTTATC	1,644	
	mcr-5R	TCATTGTGGTTGTCCTTTTCTG		
Set-2	mcr-6F-mp	AGCTATGTCAATCCCGTGAT	252	Borowiak et al. (2020)
	mcr-6R-mp	ATTGGCTAGGTTGTCAATC		
	mcr-7F-mp	GCCCTTCTTTTCGTTGTT	551	
	mcr-7R-mp	GGTTGGTCTCTTTCTCGT		
	mcr-8F-mp	TCAACAATTCTACAAAGCGTG	856	
	mcr-8R-mp	AATGCTGCGCGAATGAAG		
	mcr9-F-mp	TTCCCTTTGTTCTGGTTG	1,011	
	mcr9-R-mp	GCAGGTAATAAGTCGGTC		
Set-3	mcr-10-F	AGCCGTCTTGAACATGTGAG	744	Xu et al. (2021)
	mcr-10-R	CATACAGGGCACCGAGACTG		

### PCR results analysis

Amplified PCR products were examined on 1.5% agarose (Conda, Madrid, Spain) prepared in 100 mL of 0.5 × TBE Buffer stained with 4 μL of Nucleic Acid stain (ETB “out” Nucleic Acid, Cat. No. FYD007-200P, Yestern Biotech Co. ltd, Taiwan) for *mcr* gene. The expected bands for *mcr-1* to *mcr-10* (Table 2) were visualized and photographed under UV light using AlphaImager 2,200 (AlphaImager, United States).

### Confirmation of *mcr* (*mcr-1* and *mcr-5*) gene by sequencing

The amplified PCR products for the *mcr-1* gene of *E. coli* strains, *E. coli* E172, and *mcr-5* gene from one *Salmonella* spp. strain S283 were sent to commercial company for DNA sequencing with the same primers for Sanger sequencing (Table 2) and compared to previously reported *mcr-1* and *mcr-5* sequences in the NCBI database. The consensus sequences of *mcr-1* and *mcr-5* were obtained based on the alignment of the forward and reverse sequences using BioEdit v. 7.2 program (Hall, 1999).

### Phylogenetic analysis

The *mcr* gene sequences were used in the phylogenetic tree construction, and analysis was carried out according to Li et al. (2019). The sequences from this study and those from GenBank for the *mcr-1* and *mcr-5* were aligned separately using the MEGA X software (Kumar et al., 2018) to compare their similarities. There are four different types of *mcr-5* gene variants such as *mcr-5.1, mcr-5.2, mcr-5.3* and *mcr-5.4* has been identified in the world. We have selected partial sequences of these four *mcr-5* variants from GenBank in the NCBI database and analysed with our *mcr-5* sequence data. The phylogenetic tree for *mcr-1* and *mcr-5* was constructed with aligned sequences by the neighbor-joining method using the Kimura 2-parameter model, and Bootstrap values were calculated using 500 replicates.

### Statistical analysis

Microsoft Excel sheets (MS-2019) were used to input data, which were then uploaded into the SPSS program v. 25.0 (IBM, Armonk, NY, United States). Descriptive analysis was used to quantify the prevalence, in which level of significance was assessed using the *χ*^2^ test. For statistical significance, *p-* values less than 0.05 (*p* < 0.05) was taken into consideration.

## Results

### Frequency of *Enterobacteriaceae* isolates

In total, 627 *Enterobacteriaceae* (*E. coli*, *Salmonella* spp., and *K. pneumoniae*) isolates, including 398 isolates from meat samples from supermarkets, 153 isolates from cloacal swabs, and 76 isolates from litter samples from poultry farms, were isolated and identified (Table 3). The 398 isolates from meat samples from supermarkets were classified as *E. coli* (*n* = 258, 73.7%%), *Salmonella* spp. (*n* = 122, 34.9%) and *K. pneumoniae* (*n* = 18, 5.1%). The poultry cloacal swabs from poultry farms yielded *E. coli* (*n* = 134, 93.1%), *Salmonella* spp. (*n* = 4, 2.8%) and *K. pneumoniae* (*n* = 15, 10.4%), and litter samples from poultry farms generated *E. coli* (*n* = 44, 89.8%), *Salmonella* spp. (*n* = 14, 28.6%) and *K. pneumoniae* (*n* = 18, 36.7%). *Salmonella* spp. was highly prevalent in collected chicken meat samples (*p* = 0.000), *E. coli* in cloacal swab (*p* = 0.000), and *K. pneumoniae* was in litter samples (*p* = 0.000) (Table 3). *E. coli, Salmonella* spp., and *K. pneumoniae* isolates were confirmed by PCR showing a band size of 585 bp, 284 bp, and 130 bp, respectively.

**Table 3:** Prevalence of *Enterobacteriaceae* isolated from different sources.

Sources (Samples)	Types (Isolates)	*E. coli*	*p-*value	*Salmonella* spp.	*p-*value	*K. pneumoniae*	*p-*value
Supermarkets (*n* = 350)	Chicken meat (*n* = 398)	258 (73.7%)	0.000	122 (34.9%)	0.000	18 (5.1%)	0.000
Poultry farms (*n* = 144) (*n* = 49)	Cloacal swab (*n* = 153)	134 (93.1%)		4 (2.8%)		15 (10.4%)	
	Litter (*n* = 76)	44 (89.8%)		14 (28.6%)		18 (36.7%)	
Total (*n* = 543)	Total (*n* = 627)	436		140		51	

### Determination of phenotypic colistin susceptibility

**Figure 2:**
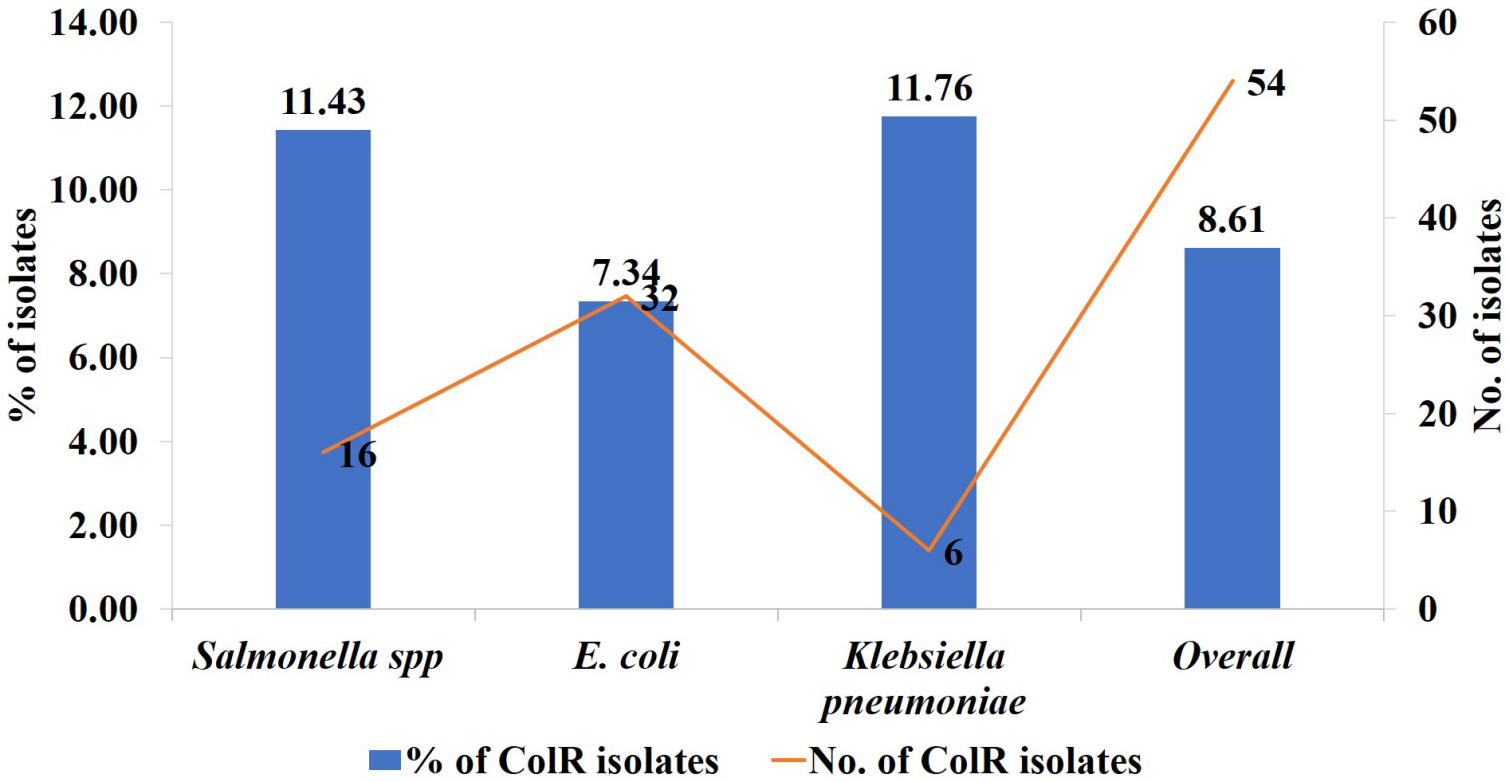
The rate trend and number of phenotypically colistin-resistant *Enterobacteriaceae* isolates, *E. coli*, *Salmonella* spp., and *K. pneumoniae.* The blue column = rate trend and red line = number of isolates, left y axis = % of colR isolates, right y axis = number of colR isolates.

Out of 627 *Enterobacteriaceae* isolates, 8.6% (*n* = 54) of isolates exhibited colistin resistance using the broth microdilution assay. Among these, 9.3% (*n* = 37) were isolated from chicken meat, 7.2% (*n* = 11) from the cloacal swab of chicken and 7.9% (*n* = 6) from the litter samples (Table 4). Overall, the phenotypic colistin resistance of the isolates from chicken meat, cloacal swabs, and litter samples was indifferent (Tables 4, *p* = 0.712). On the other hand, 54 colistin-resistant *Enterobacteriaceae* isolates were comprised of *E. coli* (*n* = 32, 7.34%), *Salmonella* spp. (*n* = 16, 11.4%), and *K. pneumoniae* (*n* = 6, 11.76%) (Figure 2).

**Table 4:** Phenotypic colistin resistance and mechanism of colistin resistance with *mcr* genes in isolates from chicken meat, chicken and litter.

Source (isolates number)	Phenotypic colistin susceptibility, (%)			*mcr* gene in colistin-resistant isolates, (%)		
	Sensitive	Resistance	*p* value	Present	Absent	*p*-value
Chicken meat (*n* = 398)	90.7%	9.3%	0.712	8.1%	91.9%	0.241
Chicken (*n* = 153)	92.8%	7.2%		27.3%	72.7%	
Litter (*n* = 76)	92.1%	7.9%		16.7%	83.3%	
Total (*n* = 627)	91.4%	8.6%		12.96%	87%	

### Detection of colistin resistance determinants, *mcr* gene variants

**Figure 3:**
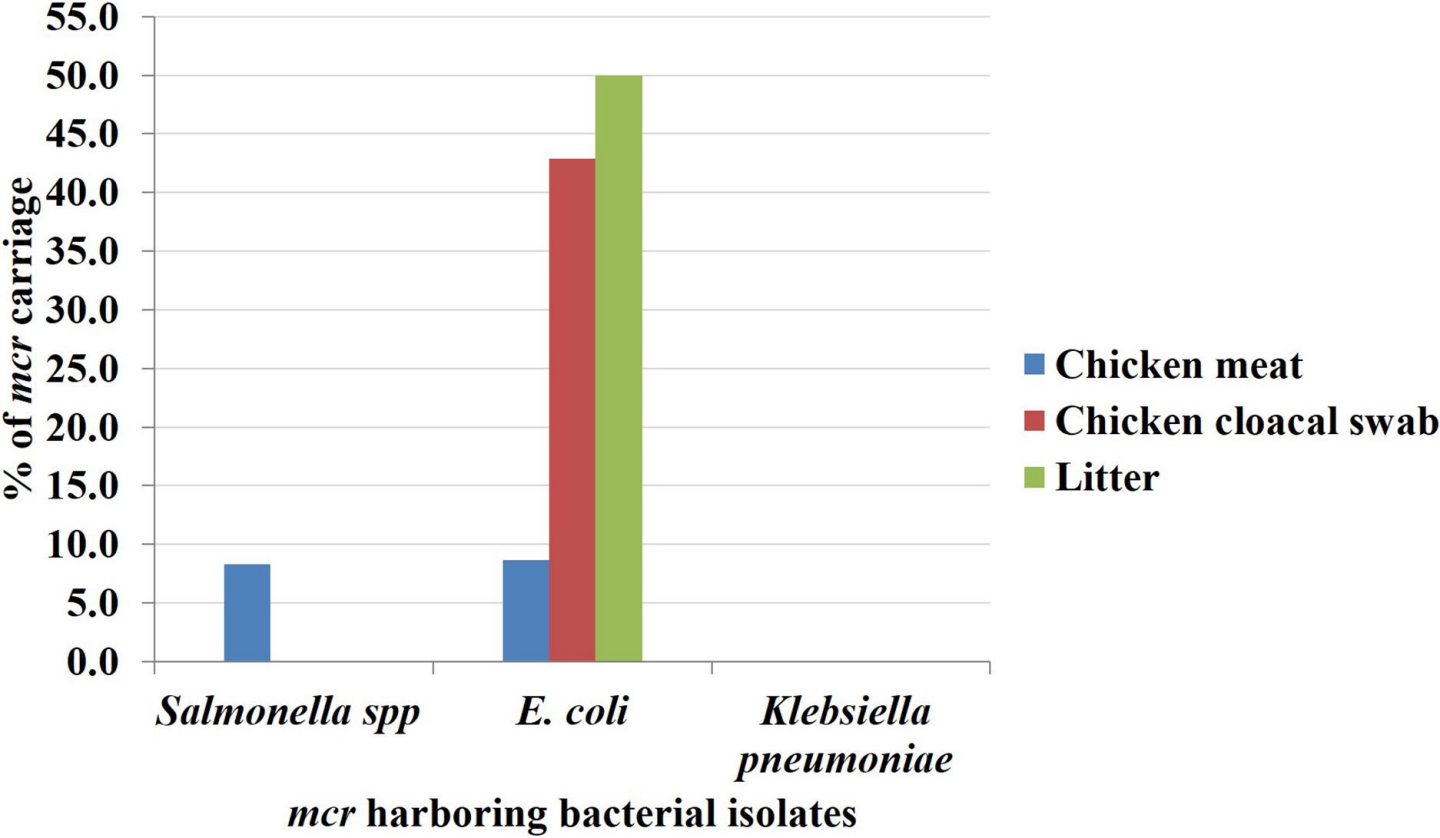
The rate of *Enterobacteriaceae* isolates harboring *mcr* gene based on sample sources. The rate of *mcr* genes was marked by blue, red and green bars to the Y-axis for chicken meat, chicken cloacal swab and litter samples, respectively.

All 54 isolated colistin-resistant (Col-R) *Enterobacteriaceae* were analyzed to observe the presence of *mcr-1* to *mcr-10.* Overall, 12.96% (*n* = 7) of colistin-resistant *Enterobacteriaceae* isolates were found possessing colistin resistance *mcr* genes comprising 8.1% (*n* = 3), 27.3% (*n* = 3), and 16.67% (*n* = 1) of Col-R isolates from the chicken meat, chicken and litter samples, respectively. Variations of colistin resistance from different sources were not statistically significant (*value of p* > 0.05) as shown in Table 4. Out of seven *mcr* harboring Col-R isolates, 11.11% (*n* = 6) and 1.85% (*n* = 1) were found with *mcr-1* and *mcr-5,* respectively. The *E. coli* isolates obtained from chicken meats, cloacal swabs and litter samples were found positive for *mcr-1*, and *Salmonella* spp. originated from the chicken meat sample was observed with *mcr-5*, whereas no *mcr* genes were observed in *K. pneumoniae* strains isolated from any of the collected samples (Figure 3). The other colistin resistance genes, including *mcr-2, mcr-3, mcr-4, mcr-6, mcr-7, mcr-8, mcr-9,* and *mcr-10* were not detected in the studied samples.

### Confirmation of *mcr gene* variants

In BLAST analysis, our studied ¬*mcr-1* sequence was found to be 100% identical to the *mcr-1* sequence (Genbank: NG_050417.1) in the NCBI database with 99% query coverage. On the other hand, our studied ¬*mcr-5* sequence was found to be 100% identical to the *mcr-5* sequence (Genbank: NG_055658) in the NCBI database with 98% query coverage.

### Minimum inhibitory concentration determination

**Figure 4:**
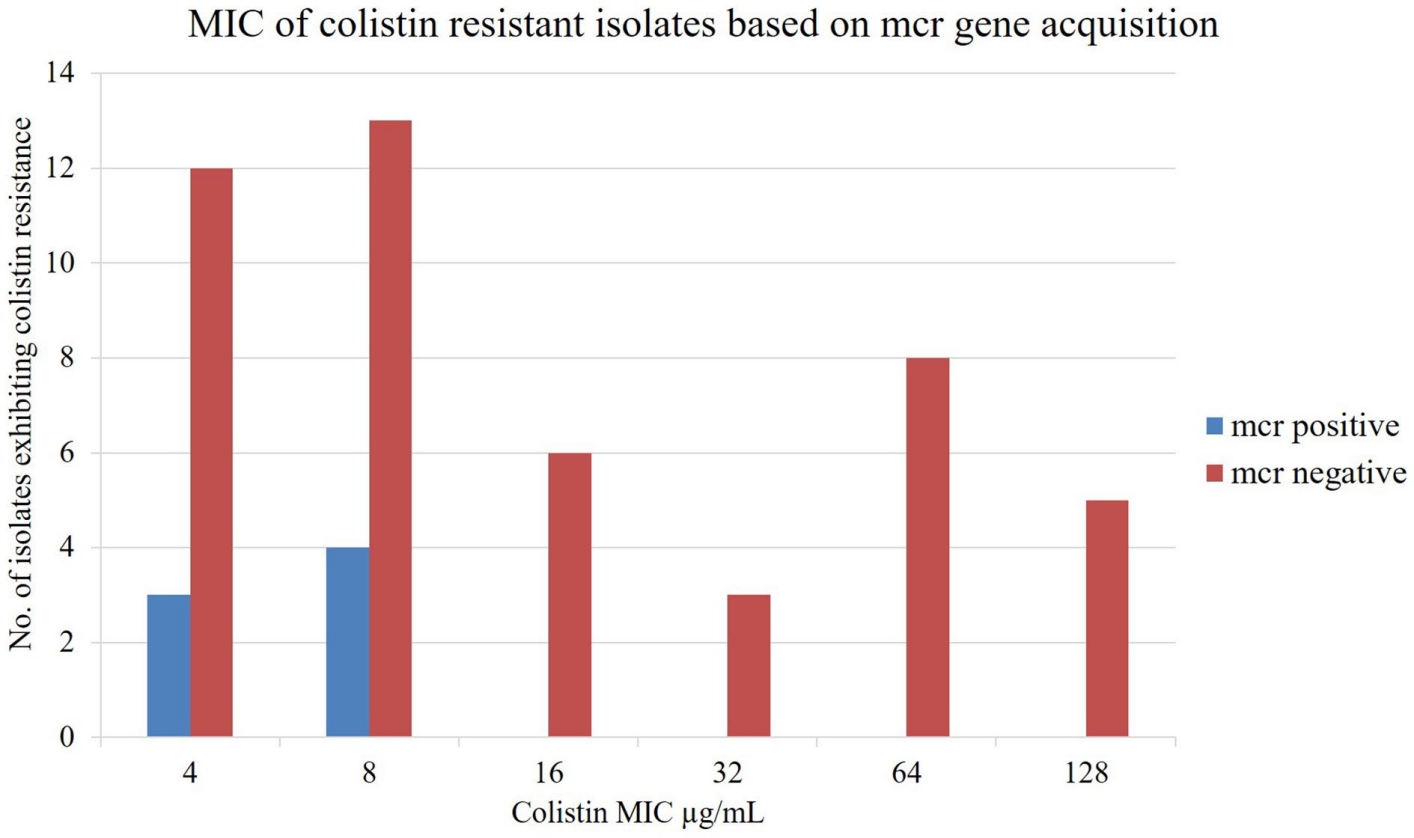
Colistin MICs of *Enterobacteriaceae* with the *mcr*-genes.

The broth microdilution test was conducted to assess the MIC value of all isolates following EUCAST guidelines, with the epidemiological cutoff value >2 μg/mL for colistin resistance. Control strains, colistin-resistant isolates *Escherichia coli* NTCC 13846 and susceptible isolate *Escherichia coli* ATCC 25922, showed growth up to 4 μg/mL and 0.5 μg/mL colistin concentration, respectively. The MIC value of isolated colistin-resistant bacteria exhibited 4 to 128 μg/mL of colistin. The *mcr*-carrying isolates were observed with MIC values of 4 and 8 μg/mL colistin (Figure 4). In contrast, *mcr* negative colistin-resistant isolates had extremely high MIC levels, 128 μg/mL colistin (Figure 4). Most of the Col-R isolates from three sources exhibited MIC values from 4 to 8 μg/mL of colistin (Supplementary Figure S1).

### Phylogenetic comparison

**Figure 5:**
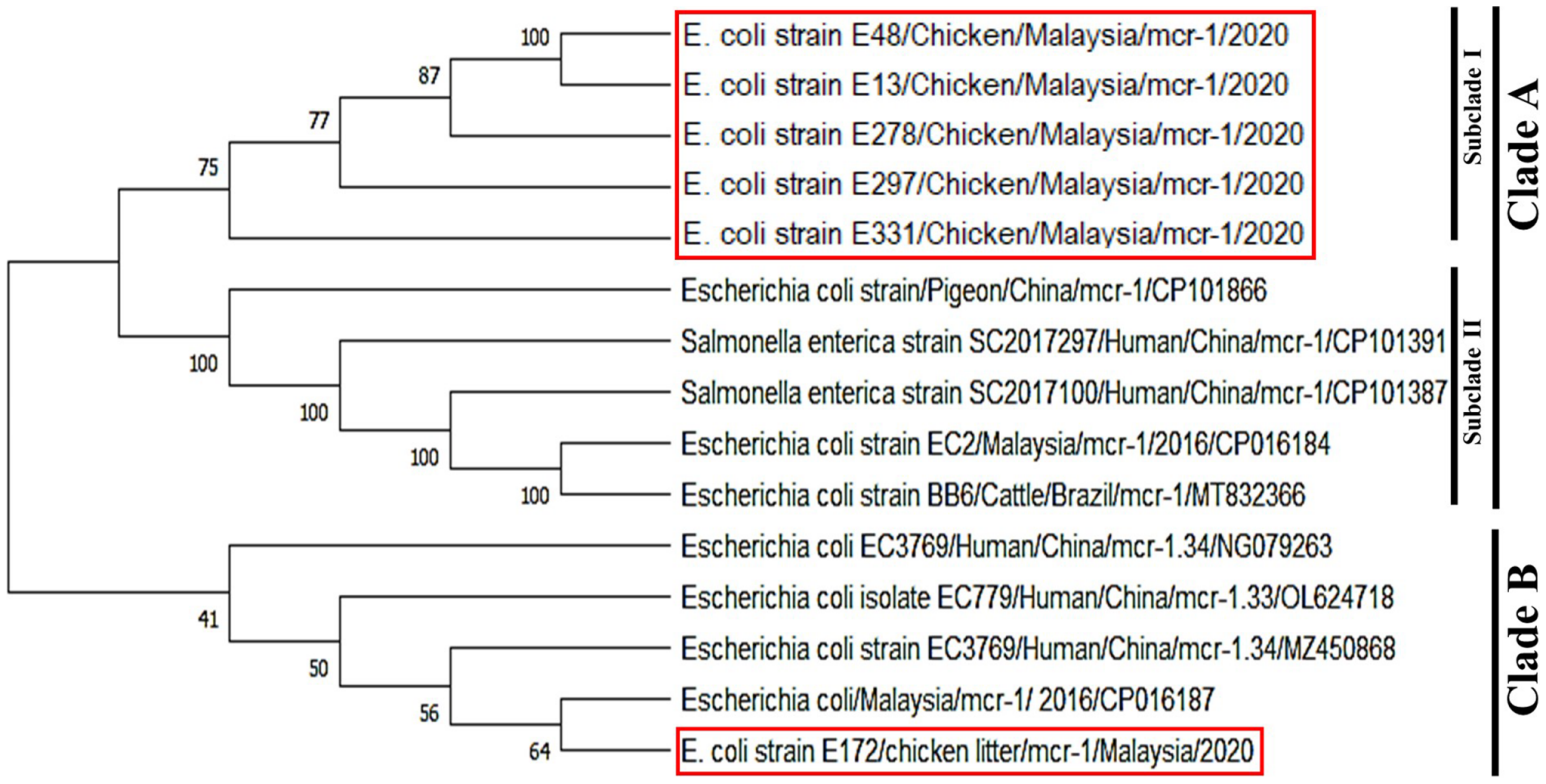
Phylogenetic tree of *mcr-1* gene showing the relationship of the partial coding regions of *mcr-1* gene sequences. The studied sequences were marked by red boxes. MEGA X was used to create the phylogenetic tree.

In the phylogeny analysis, it is observed that *mcr-1* genes in the obtained isolates were divided into two clades, named clade A, including subclade I and subclade II, and clade B. The colistin resistance *mcr-1* gene sequence from *E. coli* strains E48, E13, E278, E297, E331 recovered from cloacal swabs and chicken meat samples were clustered in subclade I. These isolates were closely related to subclade II, grouped with *E. coli* and *Salmonella* spp. strains recovered from humans and animals obtained from the NCBI GenBank database. The nucleotide sequence of the *mcr-1* gene of *E. coli* E172 obtained from chicken litter samples was very close to the previously identified isolate in Malaysia and human isolate in China. On the other hand, *mcr-1* sequences of *E. coli* isolates recovered from chicken meats, and chicken cloacal swab samples were grouped in one cluster and were found close to isolates from China and Brazil (Figure 5).

**Figure 6:**
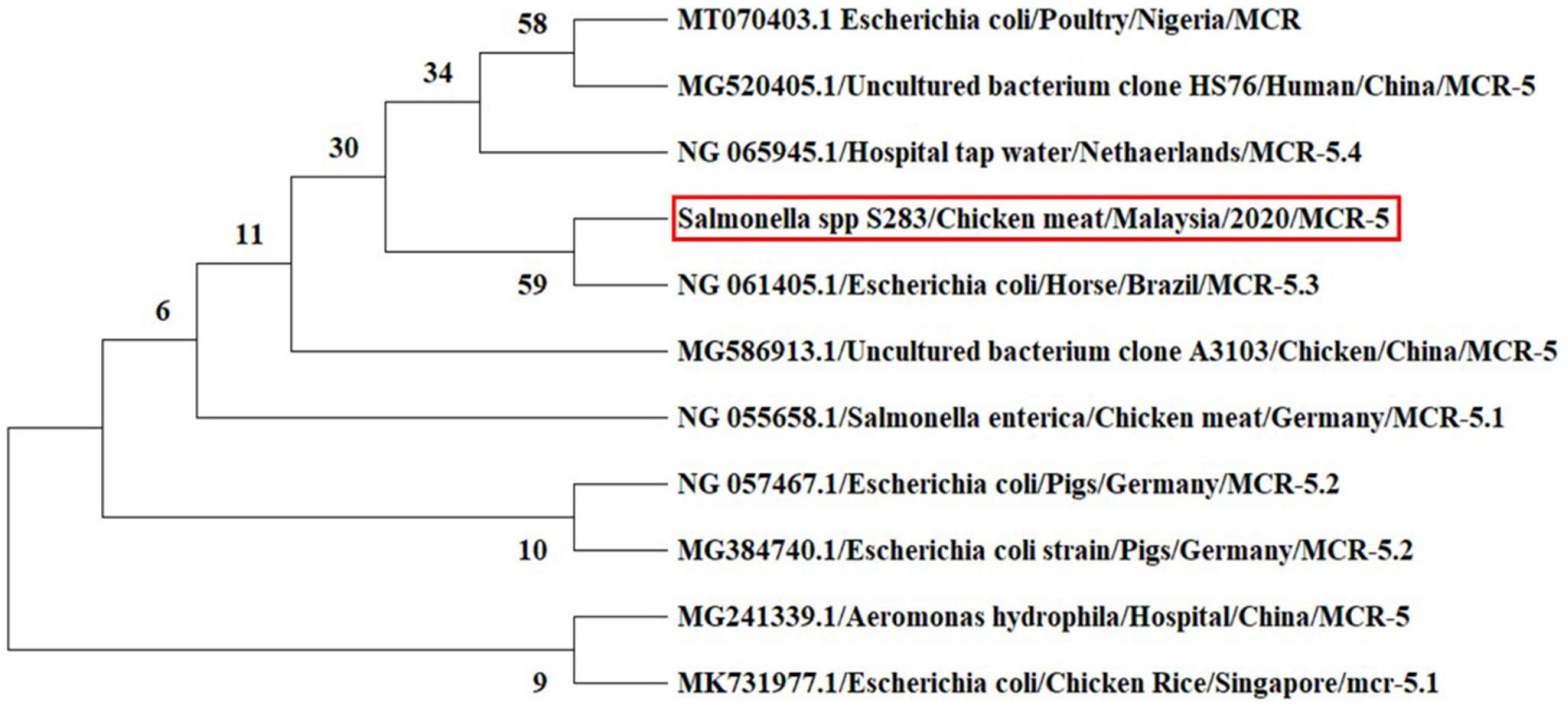
Phylogenetic tree inferred from the nucleotide sequences of *mcr-5* gene using the maximum likelihood method for distance calculations using MEGA X software. Bootstrap percentages retrieved in 500 replications are shown at the nodes. The studied sequence is marked by the red box.

In the phylogeny analysis of the *mcr-5* gene, it was revealed that the nucleotide sequence of the *mcr-5* gene of *Salmonella* spp. obtained from chicken meat has a close relation to the *mcr-5.3* gene in the *E. coli* isolate obtained from the horse in Brazil (Figure 6).

## Discussion

The most common bacteria associated with bacterial infections in chickens are *E. coli, Salmonella* spp., and *K. pneumoniae*. These microorganisms are known to cause serious health issues, which can result in increased mortality, decreased productivity, and higher costs for both disease prevention and treatment (Ibrahim et al., 2021). *Escherichia coli* isolates were the most prevalent in the collected samples, followed by *Salmonella* spp. and *K. pneumoniae* isolates. *Escherichia coli* isolates were high prevalence in chicken cloacal swabs (93.1%), *Salmonella* spp. were in chicken meats (34.9%), and *K. pneumoniae* isolates were in litter samples (36.7%). Previous studies have also reported 83 and 53.04% of cloacal swabs were found positive for *E. coli* in Malaysia and China, respectively (Suryadevara et al., 2020; Li et al., 2022). In Indonesia, 13.75% of cloacal swabs obtained from broiler chicken farms were found positive for *K. pneumoniae* (Permatasari et al., 2020). Another study in Malaysia showed that the prevalence of *Salmonella* spp. and *E. coli* from cloacal swabs obtained from broilers were 6.5 and 51.8%, respectively (Ibrahim et al., 2021). Backyard chickens in Malaysia were documented with 2.5% of *Salmonella* spp. (Jajere et al., 2019). In contrast, a much higher prevalence of *Salmonella* spp. (48%) in chicken meat samples was observed in Bangladesh (Islam et al., 2016). In Nepal, 33.33% of chicken meat samples were positive for *E. coli* (Joshi et al., 2019) which is lower than our findings, indicating that the frequency of *E. coli* in chicken meat might vary widely. Different levels of hygienic practices in various geographic regions and environmental factors, such as exposure to poor sanitation, could be the source of the variations in prevalence (Dawadi et al., 2021).

Colistin is being prescribed for the therapeutic purposes of bacterial infection in humans that are resistant to multiple drugs despite its side effects. The introduction of the colistin resistance (*mcr*) gene in bacterial strains that are already resistant to many antibiotics, creates the ineffectiveness of colistin, the last resort drug. In this study, the *Enterobacteriaceae* isolates recovered from various sources, comprising chicken meat from supermarkets and cloacal swabs and litter samples from poultry farms, were prevalent with colistin resistance and *mcr* genes. The MIC value of colistin-resistant isolates ranged from 4 μg/mL to 128 μg/mL of colistin, however, low MIC values were found in *mcr* positive isolates. In previous studies, the *mcr-1* had been documented from *Klebsiella pneumoniae,* and *E. coli* isolates in Malaysia (Yu et al., 2016; Mobasseri et al., 2019; Aklilu and Raman, 2020). The colistin resistance *mcr-1* gene is also observed in *E. coli* isolates from poultry in many countries in Asia (Trung et al., 2017; Joshi et al., 2019; Amin et al., 2020). A greater percentage of *mcr-1* (>94%) was depicted in bacterial strains from turkey and broiler feces in Germany (Irrgang et al., 2016). The colistin resistance gene (*mcr-1*) was found in 25.8% of poultry (turkey and chicken) meats from Italy (5 samples) and Germany (28 samples) but not in any samples (turkey and chicken meats) from Switzerland, Denmark, Austria, or Hungary, according to a Swiss study (Zurfluh et al., 2016). In Brazil, 19.5% of chicken meat and liver samples were positive for *E. coli* harboring *mcr-1* (Monte et al., 2017). All the colistin-resistant *E. coli* obtained from raw beef and beef products in Egypt were found positive for *mcr-1* (Sabala et al., 2021). In comparison with previous studies, a low prevalence of *mcr-1* gene in colistin-resistant isolates was found in the current research. The explanation for the low incidence in Malaysia isolates is unknown, although other resistance mechanisms, such as chromosomal alteration and modulation in the *mgrB* gene, could be involved (Chen et al., 2017).

The colistin resistance gene, *mcr-5* was detected in *Salmonella* spp. for the first time in Malaysia. There were no reports about this gene in any bacterial strains from animals or humans in Malaysia to the best of our knowledge. In this study, the *mcr-5* gene in *Salmonella* spp. was found at a very low rate. This finding is consistent with a previous report in which 2.5% (8/315) of colistin-resistant *Salmonella* spp. originated from pigs and meats (pork) in Germany (Borowiak et al., 2019), and 0.7% of human vaginal swab samples in Yangzhou in China (Zhang et al., 2018) were found to be positive for the *mcr-5* gene. The *mcr-5* gene was also observed in colistin-resistant *E. coli* strains obtained from cloacal swabs of poultry in Nigeria (Ngbede et al., 2020), veal in Belgium (Timmermans et al., 2021), pork samples in Cambodia (Pungpian et al., 2021) and poultry and pigs samples in Spain and China (García-Meniño et al., 2019). The situation is concerning because these resistant pathogens could be spread to humans through the food chain or close contact with animals (Dawadi et al., 2021).

The *mcr-1* gene was found among the isolates showing MIC values of 4 μg/mL and 8 μg/mL of colistin in this study which is consistent with the previous studies in Bangladesh and China, which showed that colistin-resistant isolates with *mcr-1* had a MIC value of 4 μg/mL, and 8 to 16 μg/mL of colistin, respectively (Islam et al., 2017; Amin et al., 2020).

In nucleotide BLAST, the sequences of the *mcr-1* gene isolated from *E. coli* and the *mcr-5* gene obtained from *Salmonella* spp. were found to be 100% identical to previously reported *mcr-1* and *mcr-5* gene sequences in the NCBI database. In the phylogenetic analysis, *mcr-1* gene sequences were closely related to previous reports, *mcr-1* gene sequence of *E. coli* isolates recovered from chicken in Malaysia (Yu et al., 2016) and *E. coli* strain isolated from human in China (Genbank_MZ450868). This suggests that *mcr-1* gene in *E. coli* has been circulating in Malaysia, which is a threat to animals and public health. The colistin resistance *mcr-5* gene was detected in *Salmonella* spp. isolate for the first time in Malaysia. The various types of *mcr-5* gene variants, including *mcr-5.1* to *mcr-5.4,* were identified in the world (Fleres et al., 2019; Ling et al., 2020). In the phylogeny, the *mcr-5* gene sequence of the current study was closely related to *mcr-5.3* gene recovered from *E. coli* isolates obtained from the horse in Brazil (GenBank database, NG_061405).

## Conclusion

The introduction and dissemination of colistin resistance with *mcr* genes in *Enterobacteriaceae* is a major worldwide issue. Colistin-resistant *Enterobacteriaceae* were observed in poultry meats and poultry farms in the present study. The *mcr-1* and *mcr-5* genes were found in colistin-resistant *E. coli* and *Salmonella* spp., respectively. The *mcr-5* has been identified in Malaysia for the first time, which could signal the advent of *mcr-5* harboring bacterial strains for infection. The existence of *mcr*-positive *Enterobacteriaceae* in poultry and poultry meat in Malaysia emphasizes the importance of proper poultry waste disposal and good hygiene practices among people who are exposed to poultry and poultry meats.

## Data availability statement

The nucleotide sequences of *mcr-1* and *mcr-5* have been submitted to the GenBank of the NCBI (accession no. OR333822, OR333835). The raw data supporting the findings of this article will be made available by the authors, without undue reservation.

## Ethics statement

The animal study was reviewed and approved by the ethical board of Universiti Putra Malaysia (UPM), Institutional Animal Care and Use Committee (IACUC) approved the research study protocol for collecting cloacal swabs from live poultry (UPM/IACUC/AUP-R091/2019).

## Author contributions

MK and ZZ: conceptualization, software, investigation, data curation, writing - original draft preparation, visualization, and acquisition. MK, ZZ, LH, NF, and NA: methodology, validation, and writing - review and editing. RK: formal analysis. ZZ: resources, supervision, and project administration. All authors contributed to the article and approved the submitted version.

## Funding

This research was funded by the UPM trust fund (grant number: 6282525) in collaboration with Bangladesh Agricultural Research Council (BARC), Dhaka, Bangladesh.

## Conflict of interest

The authors declare that the research was conducted in the absence of any commercial or financial relationships that could be construed as a potential conflict of interest.

## Publisher’s note

All claims expressed in this article are solely those of the authors and do not necessarily represent those of their affiliated organizations, or those of the publisher, the editors and the reviewers. Any product that may be evaluated in this article, or claim that may be made by its manufacturer, is not guaranteed or endorsed by the publisher.

## Supplementary material

The Supplementary material for this article can be found online at: https://www.frontiersin.org/articles/10.3389/fmicb.2023.1208314/full#supplementary-material
