# Supplementary material for: The occurrence and molecular detection of mcr-1 and mcr-5 genes in Enterobacteriaceae isolated from poultry and poultry meats in Malaysia
Source: Front Microbiol. 2023 Aug 3;14:1208314. doi: 10.3389/fmicb.2023.1208314 (PMC10435970; doi:10.3389/fmicb.2023.1208314)
Supplement: Supplementary file 1 [file Image_1.pdf]

Supplementary Material

The occurrence and molecular detection of *mcr-1* and *mcr-5* genes in *Enterobacteriaceae* isolated from poultry and poultry meats in Malaysia

Md. Rezaul Karim[1,4], Zunita Zakaria[1,5,*], Latiffah Hassan[2], Nik Mohd Faiz[3], Nur Indah Ahmad[1]

*** Correspondence:** Corresponding Author: zunita@upm.edu.my

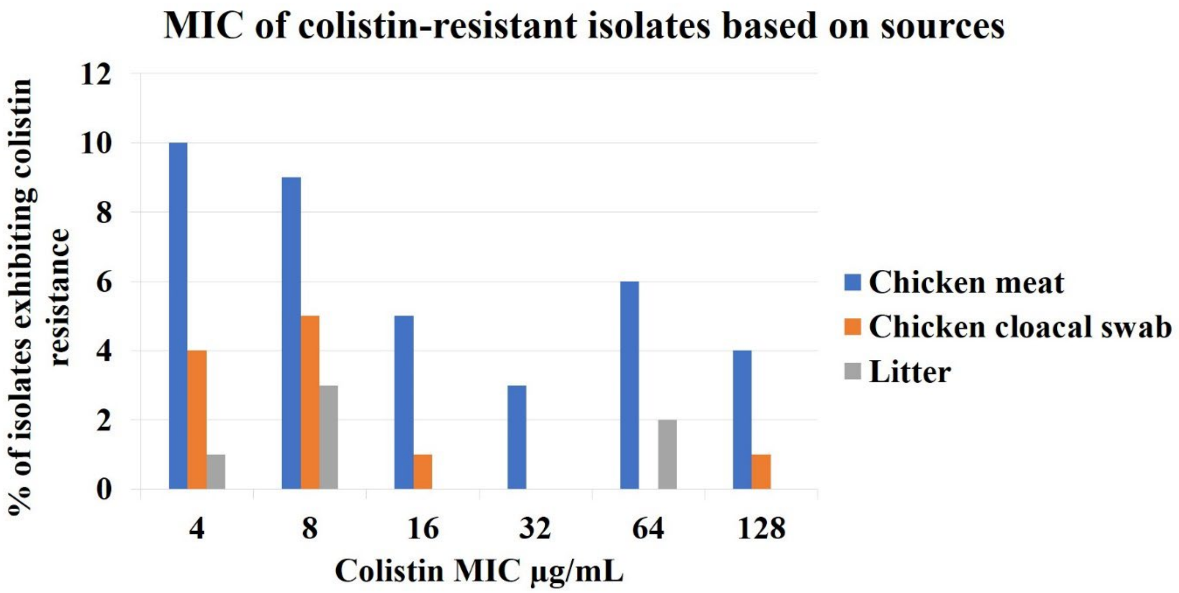

Figure S1: Colistin MICs of *Enterobacteriaceae* based on different sources
